# Nighttime surgery increases complication risk in chronic subdural hematoma: a population-based cohort study

**DOI:** 10.1007/s00701-025-06714-1

**Published:** 2025-12-03

**Authors:** Sanna Clementsson, Ali Buwaider, Jiri Bartek, Alexander Fletcher-Sandersjöö

**Affiliations:** 1 Department of Clinical Neuroscience, Karolinska Institutet, Stockholm, Sweden; 2 Department of Neurosurgery, Karolinska University Hospital, Stockholm, Sweden; 3 Department of Neurosurgery, Rigshopitalet, Copenhagen, Denmark

**Keywords:** Adverse events, Chronic subdural hematoma, Neurosurgery, Nighttime surgery, Postoperative outcomes, Surgery timing

## Abstract

**Background:**

Surgeries performed at night may carry higher risk due to provider fatigue and reduced staffing, but data from neurosurgical populations are limited. We evaluated whether nighttime evacuation of chronic subdural hematoma (CSDH) was associated with increased complications or recurrence.

**Methods:**

We conducted a retrospective cohort study of adults undergoing CSDH surgery at a tertiary neurosurgical center between 2006 and 2023. The primary exposure was nighttime surgery, defined by procedure start time. Primary outcomes were moderate-to-severe complications (Landriel-Ibáñez grade II–IV within 30 days) and CSDH recurrence (reoperation within 6 months). Multivariable logistic regression was used to adjust for confounders.

**Results:**

Of 2860 patients, 709 (25%) underwent nighttime surgery. Nighttime surgery was independently associated with an increased risk of moderate-to-severe complications (OR 1.58, 95% CI 1.04–2.37; *p* = 0.028). This risk peaked during the final hours of the night shift. Although CSDH recurrence was more common after nighttime surgery in unadjusted analysis (13% vs. 10%), this difference was not significant after confounder adjustment.

**Conclusion:**

Nighttime surgery for CSDH was associated with an increased risk of moderate-to-severe complications. When feasible, surgery should be performed during daytime hours.

## Introduction

Sleep deprivation and circadian misalignment impair cognitive and psychomotor function [14, 15], raising concerns about the safety of surgeries performed outside regular working hours [3, 21, 27, 28, 34]. In general surgery, orthopedics, and cardiac surgery, some studies have reported increased morbidity and mortality with nighttime procedures [2, 7, 36], while others have found no significant differences [31, 32]. In neurosurgery, the evidence remains limited and inconsistent [8–10, 12, 22, 26], often constrained by small sample sizes, heterogeneous procedures, and variable definitions of “nighttime”.

Chronic subdural hematoma (CSDH) evacuation offers a pragmatic model to assess the effect of surgical timing. The procedure is technically standardized, commonly performed, and frequently delayed to night hours due to scheduling constraints. To date, two studies have investigated this question in CSDH populations. A post hoc analysis of the FINISH trial (*n* = 589) found no difference in six-month functional outcomes but paradoxically reported fewer adverse events at night [26]. Gillespie et al. (*n* = 263) similarly reported no increase in complications, mortality, or CSDH recurrence [12]. However, both studies were underpowered to detect uncommon but serious events and used differing time windows to define nighttime, limiting external validity.

In light of the above, we aimed to evaluate whether nighttime surgery for CSDH is associated with increased rates of postoperative complications or hematoma recurrence, using a large, population-based cohort.

## Methods

### Study design and population

We conducted a retrospective cohort study of all patients aged ≥ 15 years who underwent surgical evacuation of a CSDH at the Karolinska University Hospital between 2006 and 2023. Cases were identified using the Orbit surgical planning system (Evry Healthcare Systems, Solna, Sweden) by the procedure code AAD10. Clinical data were extracted from the TakeCare electronic medical records system (CompuGroup Medical Sweden AB, Farsta, Sweden), and imaging data were reviewed in the Sectra PACS platform (Sectra AB, Linköping, Sweden). The study was approved by the Swedish Ethical Review Authority (EPN 2017/247 and 2013/591–31/1) who waived the need for informed consent.

Patients were excluded if they had undergone prior cranial neurosurgery within six months, had a CSDH secondary to arachnoid cysts or external hydrocephalus, or lacked data on surgical timing. These criteria are consistent with exclusion criteria in recent randomized controlled trials [4, 13, 29].

### On-call system and nighttime surgical practice

The Karolinska University Hospital is the sole neurosurgical referral center for a population of 2.4 million, providing both elective and emergency care. Neurosurgical on-call coverage follows a two-tiered system: a resident or junior specialist is present in-house, while a senior consultant is on call from home. Daytime hours are defined as 08:00–16:00 on weekdays and 09:00–16:00 on weekends, with nighttime hours spanning the remainder of the 24-h cycle. During on-call shifts, one dedicated neurosurgical operating room is available, staffed by a neurosurgical scrub nurse, an anesthesia nurse, and a neuroanesthesiologist. Surgical personnel from other specialties are available, should the need exceed these specialized resources. Computed tomography (CT), magnetic resonance imaging (MRI) and laboratory services are continuously available.

While urgent cases are prioritized, subacute cases such as CSDH may be scheduled overnight depending on clinical urgency, operating room availability, and staffing logistics. Limited daytime capacity often necessitates nighttime surgery when higher-priority procedures are scheduled during regular hours.

### CSDH treatment protocol

All patients were managed using a standardized surgical protocol throughout the study period [6]. Diagnosis was confirmed by CT or MRI. Surgery typically involved a single burr-hole craniostomy under local anesthesia, followed by irrigation with body-temperature saline and placement of a 24-h active subgaleal drain. Antibiotic prophylaxis consisted of intravenous Cloxacillin (2 g) until April 2014, after which intravenous Cefuroxime (1.5 g) became the standard. Patients with allergies received intravenous Clindamycin (600 mg). Postoperative imaging was performed at the discretion of the treating physician, generally in cases of clinical deterioration or lack of improvement. Follow-up occurred via telephone or outpatient visit within 1–2 months postoperatively.

### Exposure and outcomes

The primary exposure was nighttime surgery, defined as a procedure start time between 00:00–07:59 on weekdays and 00:00–08:59 on weekends and holidays. This definition reflects both the anticipated nadir of cognitive alertness and the formal end of the overnight shift.

Two primary outcomes were assessed:

1. Moderate-to-severe postoperative complications within 30 days, defined as Landriel-Ibáñez grades II–IV [20]. Grade I events, typically mild and self-limiting, were excluded to focus on clinically significant complications.
2. CSDH recurrence within six months, defined as reoperation for an ipsilateral hematoma.

Sensitivity analyses were conducted using two alternative nighttime definitions: 23:00–06:00, as used in the FINISH trial [26], and 20:00–07:59, reflecting a more extended nighttime period of limited staffing and reduced access to resources.

### Statistical analyses

Continuous variables were tested for normality using the Shapiro–Wilk test. As none followed a normal distribution, they are reported as medians with interquartile ranges (IQRs). Categorical variables are presented as counts and percentages. Unadjusted comparisons were made using the Mann–Whitney U test for continuous variables and Chi-square or Fisher’s exact test for categorical variables.

Associations between nighttime surgery and outcomes were evaluated using univariable and multivariable logistic regression. Covariates for the multivariable model were selected based on clinical relevance and univariable significance (*p *< 0.10), with final step-down models retaining only predictors with *p* < 0.05. Results are reported as adjusted odds ratios (ORs) with 95% confidence intervals (CIs).

To visualize the time-dependent risk of complications, we also constructed a marginal-effects logistic regression model including operative start time, Charlson Comorbidity Index (CCI), and preoperative Glasgow Coma Scale (GCS). The start time variable was sequentially varied from 00:00 to 23:59 across all patients, holding other covariates constant, to generate a smoothed risk profile across the 24-h period. This yielded a one-dimensional heat strip where color indicates the population-averaged complication risk for each minute of the day.

All statistical analyses were conducted using R (version 4.1.2), with a significance threshold of *p *< 0.05.

## Results

### Baseline characteristics

**Fig. 1:**
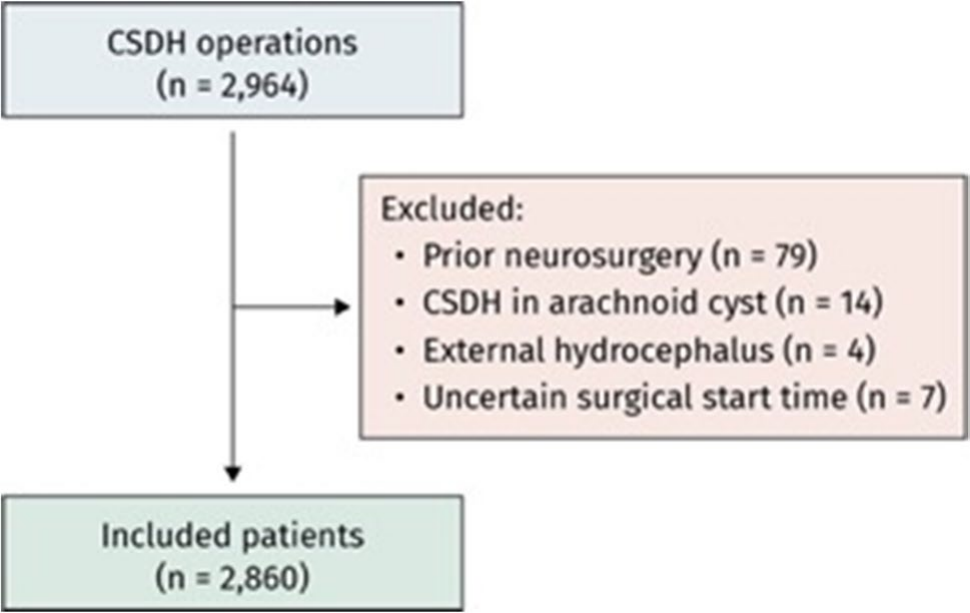
Flow-chart of the patient inclusion process

**Fig. 2:**
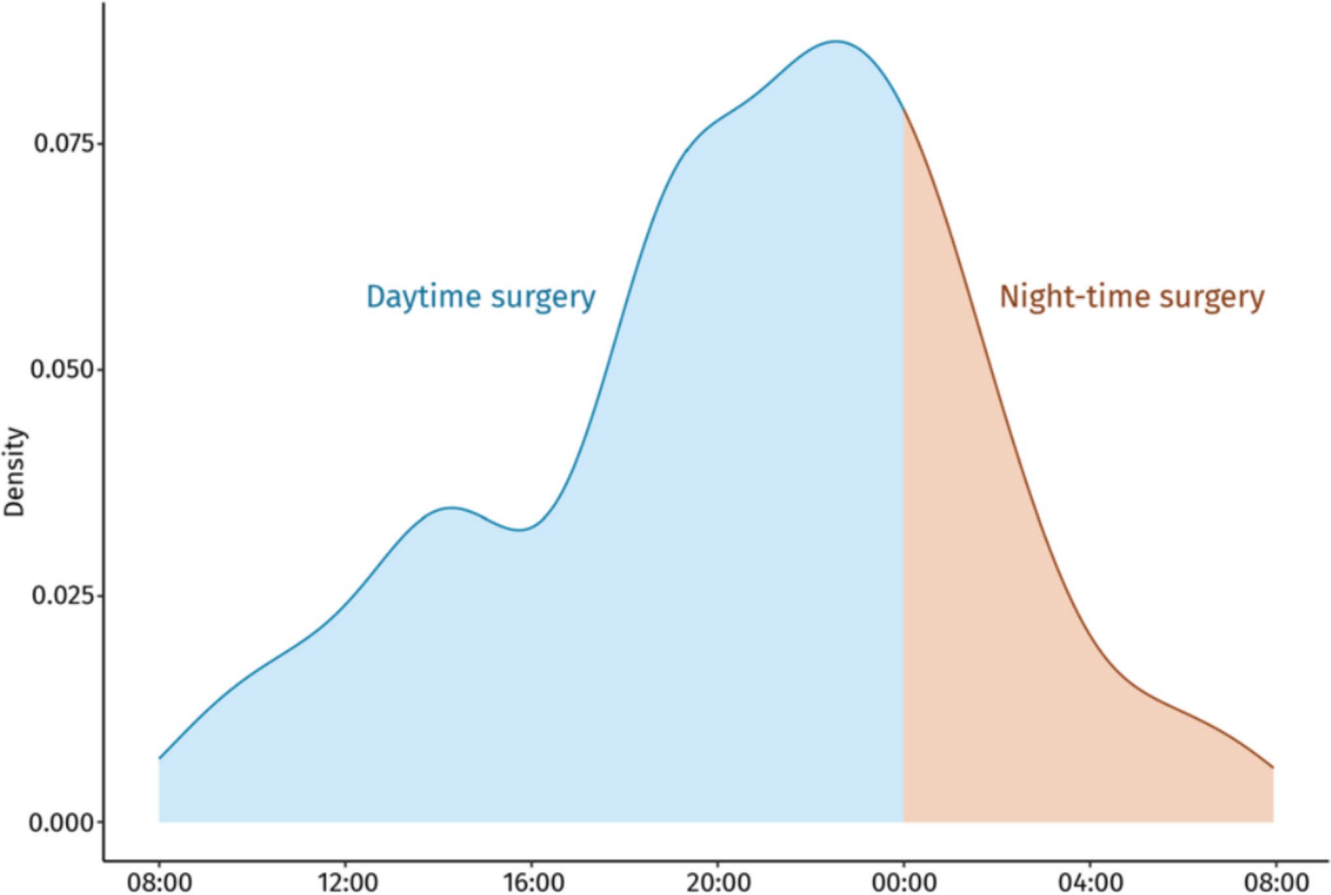
Density plot showing the distribution of surgery start times across a 24-h period, with the day structured to begin at 08:00 and extend to 07:59 the following morning. The X-axis represents the time of day, while the Y-axis indicates the density of procedures performed

Of 2964 adults who underwent CSDH evacuation during the study period, 104 were excluded: 79 due to recent cranial neurosurgery, 14 due to arachnoid cyst-associated CSDH, 4 due to external hydrocephalus, and 7 due to missing data on surgical timing (Fig. 1). The final cohort comprised 2860 patients, of whom 2151 (75%) underwent daytime surgery and 709 (25%) underwent nighttime surgery. Figure 2 illustrates the distribution of surgical start times. Procedure volume increased steadily throughout the day, dipped slightly after 16:00 (coinciding with the on-call shift change), and peaked near midnight before declining again in the early morning.

Baseline characteristics are shown in Table 1. The median age was 76 years (IQR 68–83), and 72% of patients were male, with no significant differences between groups. Preoperative comorbidity burden (CCI), neurological status (GCS and mRS), and surgical technique (burr-hole vs. mini-craniotomy) were comparable between daytime and nighttime cases. However, patients undergoing nighttime surgery had slightly larger hematoma diameters (21 mm vs. 21 mm; *p* = 0.020), more pronounced midline shift (8 mm vs. 7 mm; *p* = 0.002), and shorter operative durations (26 vs. 30 min; *p* < 0.001).

**Table 1:** Overview of the study cohort

Variable	All patients (*n *= 2860)	Day-time surgery (*n *= 2151)	Night-time surgery (*n* = 709)	*p*-value
Age (years)	76 (68–83)	76 (68–84)	76 (69–82)	0.301
Male sex	2059 (72%)	1538 (72%)	521 (73%)	0.308
Antithrombotics	1141 (40%)	841 (39%)	300 (42%)	0.129
Charlson comorbidity index	1.0 (0.0–2.0)	1.0 (0.0–2.0)	1.0 (0.0–2.0)	0.908
Preoperative GCS	15 (14–15)	15 (14–15)	15.0 (14–15)	0.932
Preoperative mRS	3.0 (2.0–4.0)	3.0 (2.0–4.0)	3.0 (2.0–4.0)	0.429
CSDH diameter (mm)	21 (17–25)	21 (17–25)	21 (18–26)	**0.020**
Midline shift (mm)	7.0 (4.0–11)	7.0 (4.0–10)	8.0 (5.0–12)	**0.002**
General anesthesia	189 (6.6%)	142 (6.6%)	47 (6.6%)	0.980
Duration of surgery (skin-to-skin) (min)	29 (21–41)	30 (21–43)	26 (19–37)	** < 0.001**
Bilateral CSDH surgery	480 (17%)	359 (17%)	121 (17%)	0.816
Surgical method				0.145
Burr-hole	2612 (91%)	1955 (91%)	657 (93%)	-
Mini-craniotomy	248 (8.7%)	196 (9.1%)	53 (7.3%)	-
Surgeons training level				0.945
Resident	2067 (73%)	1551 (73%)	516 (73%)	
Specialist	777 (27%)	584 (27%)	193 (27%)	
Missing	16	16	0	
Outcomes				
CSDH recurrence	311 (11%)	217 (10%)	94 (13%)	**0.019**
Moderate-to-severe complication	114 (4.0%)	77 (3.6%)	37 (5.2%)	0.053
Ibanez grade II	56 (2.0%)	41 (1.9%)	15 (2.1%)	0.727
Ibanez grade III	27 (0.9%)	16 (0.7%)	11 (1.6%)	0.054
Ibanez grade IV	31 (1.1%)	20 (0.9%)	11 (1.6%)	0.166

*GCS* Glasgow Coma Scale, *mRS* modified Rankin Scale, *CSDH* chronic subdural hematoma, *mm* millimeters, *min* minutes

Data are presented as median (IQR) or count (%)

Bold *p*-values indicate statistical significance (*p* < 0.05)

### Unadjusted outcomes

In unadjusted analyses, CSDH recurrence was more common in the nighttime group (13% vs. 10%; *p* = 0.019). The rate of moderate-to-severe postoperative complications was also higher at night (5.2% vs. 3.6%), though this did not reach significance (*p* = 0.053). Subgroup analysis by Landriel-Ibáñez grade showed numerically higher rates of grade III and IV events at night (Table 1).

### Multivariable analyses: complications

**Fig. 3:**
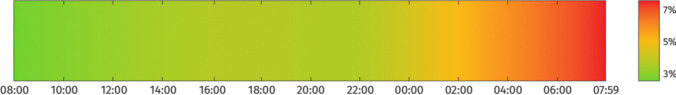
Predicted probability of moderate-to-severe complications by surgical timing. The heat strip represents the marginal predicted risk of a moderate-to-severe postoperative complication across the 24-h period. Estimates were derived from a logistic regression model including operative start time, Charlson Comorbidity Index, and preoperative Glasgow Coma Scale. The color gradient reflects the population-averaged complication risk, with green indicating lower risk and red indicating higher risk

Multivariable logistic regression identified nighttime surgery as an independent predictor of moderate-to-severe complications (OR 1.58; 95% CI 1.04–2.37; *p* = 0.028), alongside higher CCI and lower preoperative GCS (Table 2). Figure 3 illustrates complication risk across the 24-h period, with risk rising gradually overnight and peaking near the end of the night shift.

**Table 2:** Univariable and multivariable logistic regression predicting a moderate-to-severe postoperative complication

Variable	Univariable model		Final step-down multivariable model	
	**OR (95% CI)**	**p-value**	**OR (95% CI)**	***p*-value**
Night-time surgery	1.48 (0.98–2.20)	**0.054**	1.58 (1.04–2.37)	**0.028**
Age (years)	1.00 (0.99–1.02)	0.621	–	–
Male sex	1.15 (0.76–1.79)	0.533	–	–
Charlson comorbidity index	1.25 (1.13–1.38)	** < 0.001**	1.22 (1.10–1.35)	** < 0.001**
Preoperative GCS	0.79 (0.75–0.84)	** < 0.001**	0.80 (0.75–0.85)	** < 0.001**
Preoperative mRS	1.26 (1.10–1.45)	** < 0.001**	–	–
Antithrombotic treatment	1.78 (1.22–2.59)	**0.003**	–	–
CSDH diameter (mm)	1.01 (0.98–1.04)	0.476	–	–
Midline shift (mm)	1.04 (1.00–1.08)	0.067	–	–
Bilateral CSDH surgery	0.93 (0.54–1.51)	0.772	–	–
CSDH surgery using mini-craniotomy	1.01 (0.49–1.87)	0.969	–	–
Specialist training level	0.83 (0.52–1.27)	0.424	–	–

*OR* odds ratio, *GCS* Glasgow Come Scale, *mRS* modified Rankin Scale, *CSDH* chronic subdural hematoma, *mm* millimeters

Bold *p*-values indicate statistical significance (*p* < 0.05). Dashes (–) used for excluded variables in the final step-down model due to non-significant *p*-value

### Multivariable analyses: CSDH recurrence

In unadjusted analysis, nighttime surgery was associated with higher CSDH recurrence (OR 1.36; 95% CI 1.05–1.76; *p *= 0.019). However, in the adjusted model, the association attenuated and was no longer statistically significant (OR 1.28; 95% CI 0.98–1.65; *p *= 0.067). Independent predictors of CSDH recurrence instead included male sex, antithrombotic use, greater midline shift, and bilateral CSDH (Table 3).

**Table 3:** Univariable and multivariable logistic regression predicting chronic subdural hematoma recurrence

Variable	Univariable model		Final step-down multivariable model	
	**OR (95% CI)**	**p-value**	**OR (95% CI)**	***p****-value*
Night-time surgery	1.36 (1.05–1.76)	**0.019**	1.28 (0.98–1.65)	0.067
Age (years)	1.00 (0.99–1.01)	0.479	–	–
Male sex	1.54 (1.16–2.06)	**0.003**	1.46 (1.10–1.96)	**0.010**
Charlson comorbidity index	1.08 (1.00–1.16)	**0.037**	–	–
Preoperative GCS	0.93 (0.88–0.98)	**0.008**	–	–
Preoperative mRS	1.06 (0.97–1.15)	0.203	–	–
Antithrombotic treatment	1.44 (1.14–1.83)	**0.002**	1.42 (1.12–1.80)	**0.004**
CSDH diameter (mm)	1.02 (1.01–1.04)	**0.013**	–	–
Midline shift (mm)	1.04 (1.02–1.07)	** < 0.001**	1.07 (1.04–1.10)	** < 0.001**
Bilateral CSDH surgery	1.48 (1.11–1.97)	**0.007**	2.22 (1.58–3.10)	** < 0.001**
CSDH surgery using mini-craniotomy	1.05 (0.68–1.56)	0.826	–	–
Specialist training level	0.93 (0.71–1.22)	0.618	–	–

*OR* odds ratio, *GCS* Glasgow Come Scale, *mRS* modified Rankin Scale, *CSDH* chronic subdural hematoma, *mm* millimeters

Bold *p*-values indicate statistical significance (*p* < 0.05). Dashes (–) used for excluded variables in the final step-down model due to non-significant *p*-value

### Sensitivity analyses

When alternative nighttime definitions were applied, 23:00–06:00 (as in the FINISH trial) and 20:00–07:59, the associations between nighttime surgery and outcomes were no longer statistically significant after confounder adjustment. Only the primary definition used in our study (00:00–07:59 on weekdays; 00:00–08:59 on weekends/holidays) yielded a significant association with moderate-to-severe complications, supporting its clinical relevance.

## Discussion

In this large, population-based cohort study of 2860 patients undergoing surgery for CSDH, nighttime surgery was independently associated with a higher risk of moderate-to-severe complications. To our knowledge, this is the first study to demonstrate a statistically significant association between nighttime CSDH evacuation and clinically relevant complications, contributing to a growing body of evidence on circadian effects in surgical care.

Previous studies examining the impact of surgical timing have yielded mixed findings. Some have reported higher morbidity and mortality associated with nighttime procedures [2, 7, 11, 24, 36], while others found no such association [10, 12, 16, 25, 26, 31, 32]. These discrepancies likely reflect differences in procedure complexity, urgency, and institutional workflow [1, 10, 12, 16, 26]. Importantly, adverse effects of nighttime surgery may also extend beyond complications and mortality, for example several studies have reported higher rates of conversion to open surgery or prolonged hospital stays during off-hours [5, 30–32, 35].

Prior studies of specifically nighttime neurosurgery have also yielded conflicting results, often due to heterogeneous cohorts, limited sample sizes, and variable definitions of the “nighttime period” [8–10, 12, 22, 26]. In the specific context of CSDH, only two prior studies have addressed the question. The FINISH trial (*n* = 589) found no increase in complications during the night, but used a narrow time window (23:00–06:00) and was likely underpowered for rare but serious events [26]. Gillespie et al. (*n* = 263) similarly reported no increase in complications using a broader definition (20:00–08:00), but their study cohort was small [12]. In contrast, our study used a large cohort and defined nighttime based on local staffing practices, capturing periods most likely to reflect circadian fatigue and reduced support. Importantly, procedures initiated during the final hours of the night shift were associated with the highest predicted complication risk, consistent with the hypothesis that fatigue accumulates over time. This effect persisted despite no significant differences in surgeon training level or baseline comorbidity, suggesting a broader systems-level influence rather than an effect of operator inexperience.

The specific complications in the analyzed population are described in greater detail in a separate study [6]. The most common moderate-to-severe complications were subdural empyema (1.4%), surgical site infection requiring wound revision (0.3%), and cerebral herniation (0.3%). These types of complications may reflect time-sensitive vulnerabilities in perioperative care. For example, subdural empyema and wound infections could suggest lapses in sterile technique – issues potentially exacerbated during night shifts with reduced staffing and fewer experienced personnel. Cerebral herniation, while less common, may indicate delayed recognition of postoperative deterioration, which might be influenced by fatigue or limited resources at night.

### Clinical implications

Although CSDH evacuation is typically considered low-risk, our findings suggest that even routine neurosurgical procedures may carry time-dependent risks. The 1.6% absolute increase in moderate-to-severe complications associated with nighttime surgery, corresponding to a number needed to harm (NNH) of 63, is modest given the large cohort, but meaningful when considering the frequency of this procedure in neurosurgical practice. However, the relatively wide CI indicates that the true effect size may be small. While the results warrant cautious interpretation, they suggest that deferring surgery to daytime hours may reduce complication risk.

A common counterargument is that delays may compromise outcomes. Indeed, across multiple surgical specialties, earlier intervention even in non-emergent cases has been associated with benefits [1, 16, 18]. In CSDH specifically, one study suggested earlier surgery improves long-term functional outcome [17], but several others found no increase in complications, recurrence, or overall prognosis when surgery was delayed within a reasonable timeframe [19, 23, 33, 37].

Together, these findings support deferring CSDH surgery to daytime hours whenever clinical and radiological stability allows.

### Limitations

This study has several limitations. Its retrospective nature precludes definitive causal inference and is subject to selection bias and unmeasured confounding. Despite the absence of significant differences in GCS and mRS between the groups, nighttime patients may have been more clinically unstable in ways not measurable by the parameters employed. The small but significant differences in hematoma diameter and midline shift support this possibility. Surgical complexity was not directly assessed and could differ subtly by time of day. Nighttime surgery was defined based on institutional shift boundaries, which may not generalize to other centers. Furthermore, the study focused on moderate-to-severe complications (Landriel-Ibáñez grades II–IV), excluding minor events that may still impact recovery or resource use. Another limitation is that we were unable to assess the relationship between complications and functional outcome, as the absence of such an association would have strengthened the case for continued nighttime operations. Also of note, our center is a high-volume academic center with 24/7 access to imaging, anesthesia, and neurocritical care – conditions that may not be representative of smaller or less-resourced hospitals. Additionally, our hospital lacks a dedicated daytime emergency neurosurgical operating room and, consequently, clinically stable CSDH patients are often deferred to nighttime surgery. As a result, the clinical severity between the two groups is likely similar, which limits the generalizability to centers where stable patients are treated exclusively during the day.

## Conclusion

In this population-based cohort, nighttime surgery for CSDH was associated with an increased risk of moderate-to-severe complications. When feasible, surgery should be performed during daytime hours.

## Data Availability

No datasets were generated or analysed during the current study.

## Abbreviations

CCI: Charlson Comorbidity Index
CI: Confidence interval
CSDH: Chronic subdural hematoma
CT: Computed tomography
GCS: Glasgow Coma Scale
IQR: Interquartile ranges
MRI: Magnetic resonance imaging
NNH: Number needed to harm
OR: Odds ratio
